# Equity in digital healthcare – the case of Denmark

**DOI:** 10.3389/fpubh.2023.1225222

**Published:** 2023-09-06

**Authors:** Jeppe Eriksen, Mette Ebbesen, Kristina Tornbjerg Eriksen, Camilla Hjermitslev, Casper Knudsen, Pernille Bertelsen, Christian Nøhr, Ditte Weber

**Affiliations:** Department of Planning, Techno-Anthropology & Participation, Aalborg University, Aalborg, Denmark

**Keywords:** equity in digital healthcare, equity in healthcare, quintuple aim, empowerment, digital healthcare, egalitarian justice

## Abstract

As digital healthcare services are expanding in use and purpose in a Danish context so are the functionalities embedded in these, constituting citizens’ access to healthcare services and personal health data. In Denmark, the impact of inequalities in digital healthcare remains largely unexplored, making it crucial to pay close attention to this aspect as the digital transformation of the sector progresses. According to the Danish Health Act (2019), the Danish healthcare system is required to ensure easy and equal access to healthcare, high-quality treatment, coherent patient pathways, freedom of choice, easy access to information, transparency, and short waiting times for every citizen. These are focal law-based requirements influenced by the digitalisation of healthcare. Hence, based on insights from a highly digitalised country, in this case, Denmark, this paper aims to initiate a discussion on inequities in digital healthcare, address current challenges, and consider future directions by elaborating on conceptual, ethical, evidence-informed, and methodological issues linked to inequities in digital healthcare. Specifically, this paper discusses why inequities in digital healthcare in a Danish context need increased attention, how health equity is embedded in Danish legislation and how it can be approached from an ethical perspective. The central focus revolves around the essential principles of empowerment, emancipation, and equity, which are being highlighted to emphasise that the digitalisation of healthcare should actively work towards preventing and avoiding the perpetuation of healthcare inequalities. The paper concludes by discussing future directions for ensuring a more sustainable, robust, and equitable digital healthcare system.

## 1. Introduction

As one of the most digitalised healthcare sectors in the world, based on a tax-based system with universal access and a relatively well-educated population, Denmark is an interesting case to investigate, when scrutinizing equity in digital healthcare (1, 2). On a national level, the digital healthcare system in Denmark consists of services like *Sundhed.dk* (The national health portal), *Medicinkortet* (The Shared Medication Record), *Sundhedsjournalen* (The Electronic Patient Record), and *Min læge* (an app to communicate with one’s General Practitioner). These are digital services that enable citizens to access and make use of their health data, support the work of healthcare professionals (HCPs), and facilitate digital communication between HCPs and citizens (3, 4).

The digitalisation of healthcare is reflected in citizens’ use of digital technologies; hence, Danes’ use of smartphones when communicating with their General Practitioner has increased by 111% (18 to 41%) and the use of health apps has increased by 225% (20 to 66%) between 2015 and 2021. Moreover, citizens’ collection of digital health data requested by a practitioner has increased by 193% (4,3% to 12,6%) in the period from 2017 to 2021. These numbers disclose how Danes are increasingly adapting and accepting digital health technologies (DHT), this picture is also confirmed when scrutinizing numbers on citizens who do not follow their public health data online, which has declined from 42,3% in 2015 to 12,6% in 2021, indicating that almost 9 out of 10 are following their public health data online (5).

Based on the extensive digitalisation of the healthcare sector, citizens’ increased use of digital technologies and the universal nature of the Danish healthcare system, we argue that Denmark can be categorized as a *critical case* (6), which implies that challenges related to inequities in digital healthcare faced in the Danish context are likely to occur in similar or less digitalised countries now and/or in the future. Consequently, we hold the view that insights from the Danish case have global significance in trying to comprehend the dynamics of equity in digital healthcare and how it might be mitigated or prevented. However, we acknowledge that cultural, national, and societal variations, among other factors, can influence the trajectory of digital transformation in healthcare systems specific to each country.

But why discuss inequities in digital healthcare? Firstly, the concept of equity in healthcare holds a prominent position in academic and political discussions. Conversely, the attention given to equity specifically in digital healthcare is currently inadequate, which is notable, given the ongoing digital transformation of the healthcare sector. Secondly, it is crucial to ensure that the process of digitalising healthcare systems does not reproduce existing healthcare inequalities, which we argue can be avoided when developing new health technologies and digital systems if careful considerations are given to the methods employed and the underlying values embedded through design. Thirdly, healthcare and personal health data should be accessible to every citizen on a global level, which is made possible through digital platforms and devices; however, if the mission is to enable every citizen to access, use, and manage their health based on personal health data online, structures and logics constituting the digital healthcare systems need to promote genuine participation and self-management. In other words, how do we ensure that digital healthcare systems are developed according to the needs of the citizens most in need?

This paper addresses these challenges through a three-fold approach. Firstly, it explores digital health inequities from both an ethical and legislative standpoint. Secondly, it examines the fundamental concepts of empowerment, emancipation, and equity, alongside prevailing trends in healthcare policies and findings from recent research studies. Finally, exemplary cases and design approaches are highlighted, to show how the development of inclusive, fair and equitable digital healthcare systems might be achieved.

## 2. Legislation and ethics

Officially, the Danish healthcare system is characterised by government-funded healthcare with universal access (2). The Danish Health Act (2019) declares the healthcare system to promote the general health of the Danish population and to prevent and treat illness, pain, and disability in the individual patient. Further, the Danish healthcare system must meet the need for easy, timely and equal access to healthcare and information for every citizen. The aims are coherent patient pathways and a healthcare system based on freedom of choice (7).

In terms of fairness in the distribution of benefits, risks, and costs, American ethicists Tom Beauchamp and James Childress write: “*The term distributive justice refers to fair, equitable, and appropriate distribution of benefits and burdens determined by norms that structure the terms of social cooperation*” (8, p. 268). Benefits that align with citizens’ inclusive right to health, which according to the World Health Organization (WHO) primarily concerns citizens’ access to healthcare and the construction of a system that promotes equality (9).

To model a just organisation of a healthcare system, Beauchamp and Childress recommend a two-tiered model. In this model, tier 1 covers universal access to government-funded primary and acute healthcare (a decent minimum), whereas tier 2 is privately financed and covers other health issues (8). The two-tiered model incorporates egalitarian principles by ensuring universal access to basic health needs thereby protecting every citizen. While at the same time incorporating utilitarian principles of social utility, this model acknowledges, “*that society’s obligations are demanding, but not limitless*” (8, p. 293).

The Danish health system is primarily organised in line with tier 1 in Beauchamp and Childress’s model, however, the Danish system does not only cover primary and acute healthcare. Aligned with elements in tier 2, Danish citizens also have access to private healthcare providers at personal payment. Hence, the Danish healthcare system is based on elements from both tiers, but with a continuous expansion of the private sector (tier 2) over the last two decades (10). This is a development that challenges the level of *distributive justice* and the citizens’ right to equal access to healthcare services as proposed by The Danish Health Act (2019), as it benefits those who have the necessary resources to get access to tier 2 services (10). This skewness in access to healthcare requires awareness in an increasingly digitalised healthcare system; especially, as we know that early adoption of technologies is linked to individual capabilities and resources (11).

## 3. Equity, empowerment and emancipation in digital healthcare

The concept of equity and how it is substantially different from equality is essential in this context. *Equity* refers to a fair distribution or access to healthcare, whereas the related concept of *equality* refers to equal distribution or access to healthcare. Hence, *Equity* implies that digital healthcare needs to be tailored to the needs of the individual citizen, making the context, patient situation, and the freedom of choice in healthcare decision-making essential (12).

Another concept that should be clarified is digital health, which according to the WHO is “*the field of knowledge and practice associated with the development and use of digital technologies to improve health.”* (13, p. 11); an inclusive and flexible interpretation making it applicable in this paper as well. More interesting is how the digitalisation of healthcare places demands on the individual citizen to be able to handle their health with the support of digital technologies (14–16). In the Danish digital health strategy, *A Coherent and Trustworthy Health Network for All*, one of the main themes is *the patient as an active partner*. The expectations on a political level are that the digitalisation of healthcare can facilitate increased patient participation and self-management, and improve the quality of the healthcare system through timely and preventive interventions. The concept of equality is mentioned in just one section of the strategy where it utterly refers to geographical differences in healthcare delivery (17). An almost identical pattern is repeated in the recently published document describing the tasks of “The Health Structure Commission,” which are to inform the Danish government on how to shape the Danish healthcare system in the years to come. Once more, equality is mentioned in just one section, again regarding geographical differences but with an additional focus on social barriers influencing citizens’ access to healthcare (18). This almost non-existing awareness concerning inequities in healthcare and digital healthcare in Danish healthcare policies is remarkable considering how the so-called *Triple Aim’s* visions regarding improved patient experiences, better outcomes, and lower costs, historically have influenced Danish policymaking (19–21). In this context, it is noticeable that the latest additions to the Triple Aim (now referred to as the Quintuple Aim), *Clinician Well-Being* and *Health Equity* (22), have been given relatively little attention in connection to the digital transformation of the healthcare system.

The increased activation of citizens necessitates various capabilities, including the ability to locate, acquire knowledge, and adhere to health recommendations online, comprehend and utilize digital DHTs, collect and share personal health data, monitor their health metrics, communicate about their health measurements with healthcare professionals, and collaborate with family members, among other tasks (16, 23). Consequently, concepts like empowerment, eHealth literacy, emancipation, and conceptual frameworks like the 7 e’s coined by Lars Botin (24) and the 10 e’s introduced by Gunther Eysenbach (14, 16, 24, 25), need to be taken seriously and not just enacted to legitimize clinical and political actions, but implemented in a way that makes these concepts tangible, actionable, and supportive of the citizens who are most in need of the digital healthcare services.

Eysenbach, underscores the importance of the 10 e’s, where four of them represent empowerment, equity, encouragement, and education. Eysenbach argues that patients’ and HCPs’ knowledge could be improved by making better use of online information (empowerment and education) and believe that eHealth also is an opportunity to facilitate more partnership-oriented relations between HCPs and patients (encouragement) (25). Regarding equity Eysenbach writes: “*To make health care more equitable is one of the promises of e-health, but at the same time there is a considerable threat that e-health may deepen the gap between the “haves” and “have-nots.” People, who do not have the money, skills, and access to computers and networks, cannot use computers effectively. As a result, these patient populations (which would actually benefit the most from health information) are those who are the least likely to benefit from advances in information technology, unless political measures ensure equitable access for all*” (25, p. 2). This statement is more than 20 years old but as relevant as ever, which is why a continuous focus on inequities in digital healthcare and how to counter these developments still are needed.

Botin (24), introduces the 7 e’s as a way to counter inequity in digital healthcare. The idea is that “*engagement, embodiment, empathy and enactment are the means, whereas enhancement, empowerment and emancipation are the aims*” (26, p. 7). In combination with the 7 e’s Botin et al. (27), make use of the conceptual dichotomy, coined by Chris Showell and Paul Turner (28), between the Disempowered, Disengaged, and Disconnected (DDDs) citizens, who are the citizens in most need of healthcare services, and the People-Like-Us (PLUs), the empowered segment of the population, whose needs the current healthcare models are designed to cover (28). Concepts used by Showell and Turner to explain how, “*the design of personal ehealth systems may serve to accentuate the gap between privileged and disadvantaged end users and healthcare recipients, rather than improving equity of access to health care services*” (28, p. 1). Essentially, the idea is that digital healthcare systems should be designed according to the needs of those who are in most need – the DDDs (28). In Botin *et al*’s stepwise model, these concepts are integrated as the aim is to emancipate marginalized groups of citizens. The first step is to ensure that the DDDs become visible and are included as users of the digital healthcare system; the second step, the now engaged citizens need to improve their level of health literacy; the third step is where the citizen is empowered to a degree that allows the individual to actively engage with the healthcare system in a partnership relation; and fourth step, is the emancipation of the citizen, which “involves self-reflection and in-depth knowledge of one’s health condition, high levels of health and eHealth literacy, and detailed understanding of the how’s and the why’s of the system” (27). Hence, the stepwise model can be used as a guideline, when discussing how to include marginalized citizens and promote health equity.

The focus on the individual’s competencies versus the design of the systems constituting the digital healthcare system is also captured very well in this statement by the Danish Professor and consultant Morten Sodemann, “*some, even believe that then you need to improve the patients’ health literacy, enabling them to keep up – not a word about how it should be the other way around: The healthcare system should improve their patient competencies*” (29, p. 3). Interestingly, this quote mirrors two types of empowerment, one is *patient empowerment*, which according to HCPs concerns, dissemination of information, decision-making, and disease management, holistic and trusting nurse–patient relations; support groups and supportive care; and a feeling of independence, control, and autonomy (30). The other type of empowerment, the one that Sodeman believes the healthcare system is in short supply of, is the *critical approach*, where the aim is to develop the *critical consciousness* of not only individuals, but entire oppressed groups of a population to give them a voice, political influence, and enable them to change structures and emancipate themselves (31). The former approach emphasises the development of the individual’s ability to self-manage one’s health or disease, which is linked to the individual’s health behaviour and lifestyle; however, according to Signild Vallgårda (12), an individual’s health status should also be assessed through the inclusion of other health determinants like genetics, upbringing, work environment, social conditions, and other external elements. In other words, it is not enough that change comes from within the individual, if decision-makers are serious about building sustainable and robust digital healthcare systems that promote equity, then alterations, modifications, and optimisations of the structures comprising the digital healthcare system need to be tailored to the life situation, needs and preferences of those individuals who are most in need (12, 27, 28).

This emphasis on both the individual competencies and the adaptation of the healthcare systems is mirrored in Monkman and Kushniruk’s (14) coining of eHealth literacy, as they underscore that the usefulness and useability of eHealth applications and systems depend not only on the consumer’s level of eHealth literacy but also on the system’s demands on eHealth Literacy. As a result, the education of consumers to raise their eHealth literacy level should happen in parallel with the development of health information systems that minimize the level of eHealth literacy needed to use these systems (14). Hence, this approach to eHealth literacy serves as an example of how increased equity in digital healthcare may be achieved by focusing, not only on the individual citizen but also on the development and design of the systems that mediate the healthcare services.

Inequities in healthcare manifest due to several factors, as described in the PROGRESS PLUS framework, which is an abbreviation for Place of residence, Race/ethnicity/culture/language, Occupation, Gender/sex, Religion, Education, Socioeconomic status, and Social capital (32). Notably, divisions linked to these factors seem to be reproduced in digital healthcare. Hence, in a scoping review conducted by the WHO evidence shows that DHTs to a larger degree are used by younger, white ethnic, English speakers, with higher education and economic status, living in an urban area, compared to individuals from ethnic minorities with language barriers, lower education, and economic status from rural areas (33). Similar tendencies are disclosed by inquiries made by Danish authorities who find that citizens’ age, education, employment, and civil status explain variations in health statuses (34), whereas social and physical determinants to work environment and labour market connection affect inequities in healthcare (35, 36). Despite these investigations of inequities in healthcare, none of the ministerial reports scrutinizes how digitalisation affects existing inequities. This observation is significant as it suggests that decision-makers may not fully comprehend the seriousness of this issue. This is an intriguing finding since Danish researchers have demonstrated that individuals with higher education levels tend to utilize the national health portal more frequently and experience greater ease in navigating digital health systems, in contrast to those with lower education levels (37).

## 4. Interventions promoting equity in digital healthcare

To complement the theoretical, conceptual, and ethical perspectives on inequities in digital health, this section shortly unfolds experiences and participatory approaches that are useful when developing sustainable, responsible, and useful DHTs meant to promote equity in digital healthcare. A necessary strategy acknowledged by the WHO, since “*Inclusive and participatory design approaches, such as co-design and co-production, are required to ensure that DHT approaches have usability and meet needs across population groups*” (33, p. viii).

In the same vein, Veinot et al. (38), underscore the importance of preventing *intervention-generated inequalities*, and they suggest that every choice made when designing health information evaluations should take equity into account. Hence, when choosing relevant independent variables, outcome variables and whom to recruit, one should ensure that factors associated with equity issues are considered and deliberately integrated into the study design (38).

One approach to participatory design is User Innovation Management (UIM), which basically is a user-centred design process. The advantage of the UIM method is the explicit focus on the selection of participants, how, who, and why should specific individuals and groups be part of the design process. Moreover, the user context and intended outcomes are considered continuously throughout the design process to ensure that concepts and sketches are developed according to the needs of the users (39).

Decentralized Patient-centred Clinical Trials (PACT) and MOVE are two Danish projects that exemplify how these types of user-centred approaches are applicable in a digital healthcare context. PACT is a large national project which seeks to improve equity in digital healthcare, by bringing clinical trials closer to the patient and making healthcare more accessible using digital solutions. This is achieved by creating a national IT platform where patients and healthcare providers can view and access all clinical trials and by strengthening the infrastructure around decentralizing clinical trials in Denmark (40, 41). MOVE is a mobile app that supports people in forming social relations around exercise in a high-health-risk residential area. MOVE was developed by using the UIM method in collaboration with so-called marginalized citizens and HCPs (42).

Other examples of how user perspectives are used to promote equity in digital healthcare are the DiGi project, where digital social relations among youth living with cognitive disabilities are examined to make sure that the needs of the target group are integrated into future digital innovations (43), and the EXOTIC project, where people living with tetraplegia are interviewed through the use of design games ensuring that an upper-limb exoskeleton is designed according to their needs (44).

Conclusively, it should be underscored that inequities in digital healthcare can, and according to law and ethics, should be mitigated and/or prevented. Currently, we are creating standards and infrastructures that deliberately exclude the perspectives of those, who are most in need of the services offered by the digital healthcare system; resulting in a narrow perception of reality or as Geoffrey C. Bowker and Susan L. Star declare, “*We will see the blind leading the blind. This blindness occurs by changing the world such that the system’s description of reality becomes the true*” (45, p. 49). This reductionistic constitution of reality is what needs to be countered by empowering and emancipating marginalized groups. As a result, one of the main missions in the development of future digital healthcare systems should be to make the invisible citizens visible and help the “blind” decision-makers and developers “see”; that is if we are to achieve a sustainable and robust digital healthcare system that promotes equity in health. A good place to start is by discussing the impact of digital healthcare on health inequities.

## Data availability statement

The original contributions presented in the study are included in the article/supplementary material, further inquiries can be directed to the corresponding author.

## Author contributions

JE, ME, KE, CH, CK, PB, CN, and DW: conceptualization and investigation. JE: formal analysis. JE, ME, and DW: writing – original draft. JE, ME, DW, KE, and CH: writing – review and editing. JE: supervision. All authors contributed to the article and approved the submitted version.

## Conflict of interest

The authors declare that the research was conducted in the absence of any commercial or financial relationships that could be construed as a potential conflict of interest.

## Publisher’s note

All claims expressed in this article are solely those of the authors and do not necessarily represent those of their affiliated organizations, or those of the publisher, the editors and the reviewers. Any product that may be evaluated in this article, or claim that may be made by its manufacturer, is not guaranteed or endorsed by the publisher.
